# Monitoring Great Ape and Elephant Abundance at Large Spatial Scales: Measuring Effectiveness of a Conservation Landscape

**DOI:** 10.1371/journal.pone.0010294

**Published:** 2010-04-23

**Authors:** Emma J. Stokes, Samantha Strindberg, Parfait C. Bakabana, Paul W. Elkan, Fortuné C. Iyenguet, Bola Madzoké, Guy Aimé F. Malanda, Brice S. Mowawa, Calixte Moukoumbou, Franck K. Ouakabadio, Hugo J. Rainey

**Affiliations:** 1 Global Conservation Program, Wildlife Conservation Society, New York, New York, United States of America; 2 Congo Program, Wildlife Conservation Society, Brazzaville, Republic of Congo; 3 Ministère de l'Economie Forestière, Brazzaville, Republic of Congo; 4 Brazzaville, Republic of Congo; University of California, United States of America

## Abstract

Protected areas are fundamental to biodiversity conservation, but there is growing recognition of the need to extend beyond protected areas to meet the ecological requirements of species at larger scales. Landscape-scale conservation requires an evaluation of management impact on biodiversity under different land-use strategies; this is challenging and there exist few empirical studies. In a conservation landscape in northern Republic of Congo we demonstrate the application of a large-scale monitoring program designed to evaluate the impact of conservation interventions on three globally threatened species: western gorillas, chimpanzees and forest elephants, under three land-use types: integral protection, commercial logging, and community-based natural resource management. We applied distance-sampling methods to examine species abundance across different land-use types under varying degrees of management and human disturbance. We found no clear trends in abundance between land-use types. However, units with interventions designed to reduce poaching and protect habitats - irrespective of land-use type - harboured all three species at consistently higher abundance than a neighbouring logging concession undergoing no wildlife management. We applied Generalized-Additive Models to evaluate *a priori* predictions of species response to different landscape processes. Our results indicate that, given adequate protection from poaching, elephants and gorillas can profit from herbaceous vegetation in recently logged forests and maintain access to ecologically important resources located outside of protected areas. However, proximity to the single integrally protected area in the landscape maintained an overriding positive influence on elephant abundance, and logging roads – even subject to anti-poaching controls - were exploited by elephant poachers and had a major negative influence on elephant distribution. Chimpanzees show a clear preference for unlogged or more mature forests and human disturbance had a negative influence on chimpanzee abundance, in spite of anti-poaching interventions. We caution against the pitfalls of missing and confounded co-variables in model-based estimation approaches and highlight the importance of spatial scale in the response of different species to landscape processes. We stress the importance of a stratified design-based approach to monitoring species status in response to conservation interventions and advocate a holistic framework for landscape-scale monitoring that includes smaller-scale targeted research and punctual assessment of threats.

## Introduction

It is widely recognized that effective conservation planning needs to consider both the ecological requirements of wildlife as well as the economic needs of people [1], [2]. Protected areas continue to form the cornerstone of biodiversity conservation, but for many wide-ranging or migratory species, strict protection is often not possible over large spatial scales. Under this scenario has evolved the concept of the conservation landscape [3]; a mosaic of protected areas embedded in a matrix of multiple land-use types employing a variety of different management strategies. Incorporation of ‘biodiversity friendly’ land-use practices into actively managed buffer zones can not only protect critical habitats for a variety of different species [4], but also contribute to the long-term conservation value of core protected areas [1]. Monitoring the status of wildlife under different management strategies and evaluating the success of these strategies in meeting conservation or policy objectives is of increasing interest to practitioners managing biodiversity at the landscape scale [5]–[7]. In this context the design of wildlife monitoring programs is challenging. Firstly, landscapes are dynamic, with an inherent spatial and temporal heterogeneity in the natural and human systems that must be addressed by sampling design in order to provide un-biased estimates of wildlife abundance at ecological meaningful scales [8]–[10]. Secondly, monitoring programs need to be designed with adequate estimator precision and statistical power to detect a desired change, for example an increase in species abundance in response to interventions [11]–[13]. Finally, a myriad of complex interactions between landscape systems confound our ability to make accurate predictions about the response of wildlife to management actions. Nevertheless, monitoring programs targeted at evaluating different hypotheses about wildlife responses to management, are an integral part of an adaptive management process (*sensu* [14]); monitoring programs in and of themselves should provide information with which to refine these predictions as part of an iterative learning process [10], [15], [16].

The forests of the Congo Basin are one of the world's last remaining tropical wildernesses [17] and a top priority for biodiversity conservation [18], harbouring several globally threatened large mammal species such as forest elephants *Loxodonta africana cyclotis*, western gorillas *Gorilla gorilla gorilla* and common chimpanzees *Pan troglodytes troglodytes*. In spite of legal protection across their range, recent reports on all three of these species suggest populations are declining rapidly through a combination of poaching and, in the case of great apes, disease [19], [20]. Over the past two decades, commercial poaching of all three species has been exacerbated by the rapid expansion of industrial logging activities and infrastructure, including roads [20]–[23]. Commercial logging is prevalent throughout much of the Congo Basin, with over 30% of native forest allocated to logging concessions, compared to only 12% under protection [24]. More than 50% of the current range of western gorillas and sympatric chimpanzees for example is estimated to lie in active logging concessions [25]. Biodiversity management at the landscape level is a relatively new concept in the Congo Basin and the creation of baseline datasets to evaluate the efficacy of conservation strategies is only just beginning. A strategic objective of current international conservation efforts, coordinated through the Congo Basin Forest Partnership (CBFP), is to evaluate the effectiveness of different management approaches – i.e. protection, sustainable management of commercial logging concessions and community-based natural resource management - in priority landscapes through a suite of biodiversity indicators that include wide-ranging, charismatic or endangered species [26]. The technical challenges of designing management-oriented landscape-scale wildlife monitoring programs for large-bodied, rare or cryptic species are compounded in the Congo Basin by the logistical challenges of accessing vast and remote forests with low technical capacity, thinly stretched budgets and, in many cases, armed conflict [27], [28]. It is therefore unsurprising that in the Congo Basin there exist few examples of large-scale wildlife surveys (but see [19], [29], WCS-Gabon unpubl.) or systematic landscape-scale monitoring efforts, with which to evaluate the status of wildlife populations or the success of different management strategies with respect to key wildlife targets.

**Figure 1:**
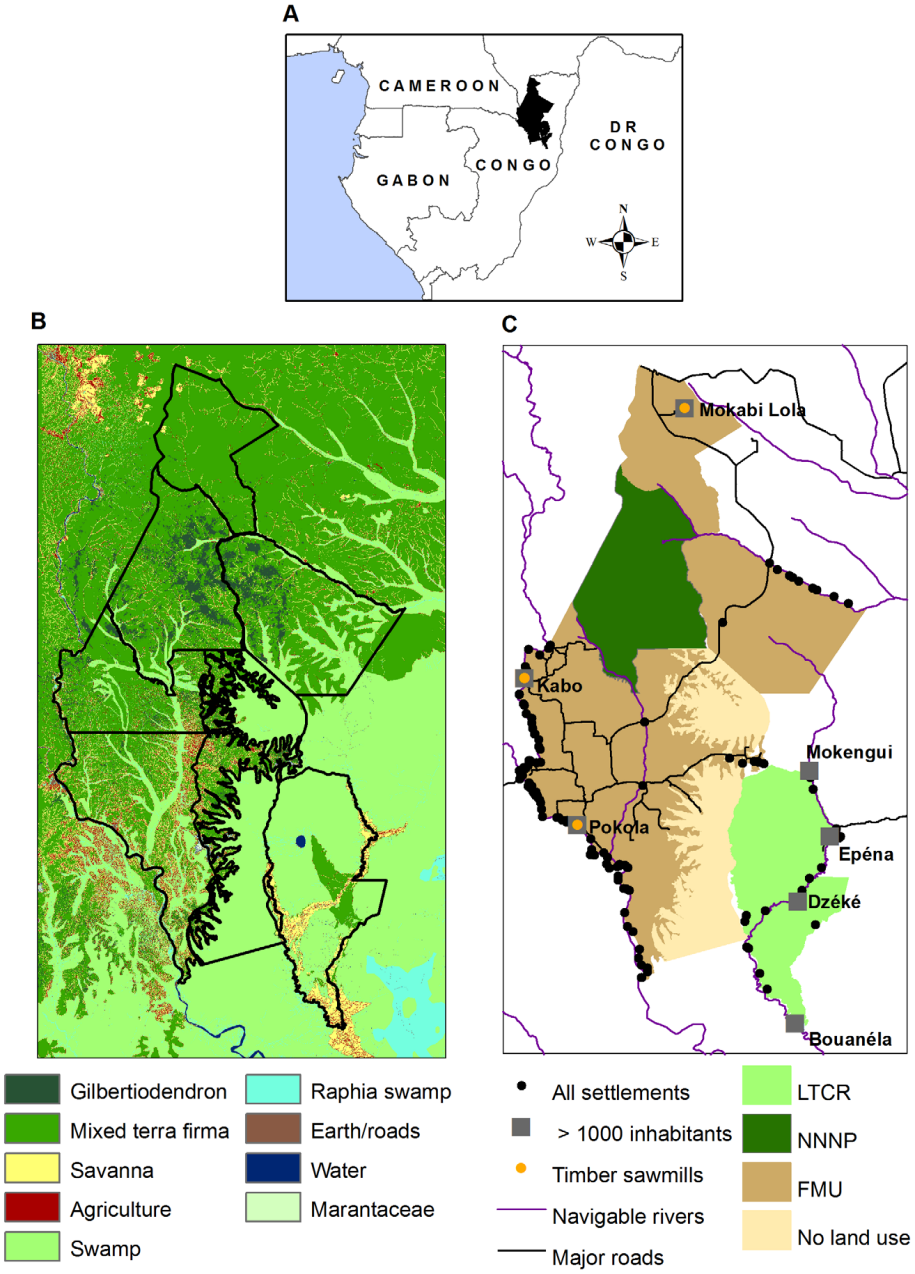
The Ndoki-Likoula Conservation Landscape. A - Geographic location, B - Main vegetation types, and C - Land-use types and human access features.

This paper presents base-line data from a landscape-level wildlife survey conducted in northern Republic of Congo (abbreviated here as Congo). Northern Congo harbours one of the largest remaining populations of forest elephants [19], and the largest remaining populations of western gorillas and chimpanzees in Africa [30], [31]. At the same time, it has one of the fastest rates of expansion of mechanized logging in the Congo Basin, with the rate of logging road construction increasing four-fold between 1990 and 2000 [24]. The Ndoki-Likouala Conservation Landscape in northern Congo encompasses two protected areas surrounded by several commercial logging concessions (**Figure 1**). Since 1991, the Wildlife Conservation Society (WCS), in collaboration with the Government of Congo and international public and private-sector partners, has established three major site-based conservation projects across the Ndoki-Likouala landscape, implementing three different wildlife management strategies across contiguous zones; 1) integral protection of wildlife and their habitat in a core protected area – the Nouabalé-Ndoki National Park (NNNP), 2) community-based conservation and management of wildlife and other natural resources in and around the swamp forests of the Lac Télé Community Reserve (LTCR), and 3) wildlife management and conservation in several surrounding commercial logging concessions or Forestry Management Units (FMUs) [32], [33]. These three strategies are implemented with the combined goal of conserving ecologically functional populations of forest elephants, great apes and other focal species across the Ndoki-Likouala landscape. This is achieved through a ‘landscape-species approach’ (*sensu* [3]), which maps spatially-explicit ecological requirements for a suite of conservation targets (‘landscape species’) and, based on their overlap with human land uses, identifies key threats to be addressed by conservation action [3], [34], [35]. The Ndoki-Likouala monitoring program was developed with the primary objective of evaluating the impact of different management strategies on the density and abundance of landscape species.

In this paper we demonstrate the application of a large-scale (28,000km^2^) monitoring program designed to evaluate the impact of different management strategies on three landscape species of conservation concern – forest elephants, western gorillas and chimpanzees. We present the first year of data from this program and examine the extent to which our survey design and estimation approaches succeed in meeting the program's objective. Specifically, our aims are three-fold: firstly, we assess the status of these three species in different management units through the application of design-based stratified distance-sampling methods. Management units are defined here as discrete areas, typically defined spatially by government decree and operating under a clearly defined management authority and/or land-use type (**Table 1**). Different management units are subject to varying degrees of wildlife management (varying in the type of interventions and stage of implementation) and impacted by varying degrees of human pressure (**Table 1**). Secondly, we apply Generalized Additive Models to examine a series of *a priori* hypotheses governing the spatial relationships between the distribution of these three species, human activities (including management strategies) and ecological variables (**Table 2**) and evaluate the utility of such models in enhancing our knowledge of the landscape system and improving the precision of abundance estimates. Finally, we integrate these findings in order to provide practical recommendations for the implementation of monitoring programs designed to evaluate and inform the effectiveness of conservation landscape interventions.

**Table 1:** Socio-economic and management features of the Ndoki-Likouala Conservation Landscape.

Land Management unit	Area (km^2^)	Human pop.	Road density (km/km^2^)	Primary land use	Start of logging activities	Start of wildlife mgmt.	Managmt. partners^1^	Managmt. plan status	Primary wildlife management interventions	Direct threats addressed^2^
NNNP	4,190	0	0	Protection	Not logged	1991	WCS/MEF	Adopted	Law enforcement	Poaching
Kabo FMU	2,870	4,220	1.07	Logging	1968	1999	CIB/WCS/MEF	Adopted^3^	Law enforcement, Roadblocks, zoning	Poaching, habitat loss/degradation
Pokola FMU	4,510	16,300	1.08	Logging	1968	2000	CIB/WCS/MEF	Underway	Law enforcement, Roadblocks, zoning	Poaching, habitat loss/degradation
Loundougou FMU	4,230	2,690	0.20	Logging	2005	2001	CIB/WCS/MEF	Underway	Law enforcement, Roadblocks, zoning	Poaching, habitat loss/degradation
Toukoulaka FMU	2,080	1,360	1.72	Logging	1992	2000	CIB/WCS/MEF	Underway	Law enforcement, Roadblocks, zoning	Poaching, habitat loss/degradation
Mokabi	2,670	1,980	0.12	Logging	2000	-	Rougier-MOKABI	Initiated	None^4^	None
Bailly/Bodingo swamps	3,770	0	0.02	-	Not logged	-	-	-	Law enforcement, community mgmt.^5^	Poaching^5^
LTCR	4,380	14,750	0.001	CBNRM^6^	Not logged	2000	WCS/MEF	Underway	Law enforcement, community mgmt.	Poaching

1 WCS (Wildlife Conservation Society); MEF (Ministry of Forest Economy); CIB (Congolaise Industrielle des Bois) - a subsidiary company of the Danish timber group DHL; Rougier-MOKABI, a subsidiary timber company of the French timber group Rougier SA.

2 Refers to threats to focal species of this paper: elephants, gorillas and chimpanzees.

3 At the time of the surveys, Kabo was the first concession in the Congo Basin to have been awarded Forest Stewardship Council (FSC) certification in May 2006 (Tropical Forest Trust, 2006. First forest in the Congo to achieve highest international standard of good management. http://www.tropicalforesttrust.com/news-detail.php?newsid=47.

4 At the time of the surveys there were some anti-poaching patrols along the northern border of the NNNP/southern sector of Mokabi as part of the NNNP anti-poaching program.

5 Much of this unit is contiguous with the LTCR and gains some benefits from community management and anti-poaching interventions in LTCR. Correspondingly, the communities in LTCR also visit the Bailly for livelihoods activities, although they are not resident in the Bailly.

6 Community-Based Natural Resource Management.

**Table 2:** Hypotheses and predictions tested for spatial distribution of ape nest and elephant dung counts.

Covariate	Species^1^	Hypothesis	Prediction	Supported (this study)
**Vegetation type**	*E,G*	Attracted to forests rich in herbaceous food plants	Higher density found in dense understory mixed-forests, swamp and secondary forests	Partially^2^
	*C*	Attracted to forests rich in mature fruiting trees	Higher density in primary closed canopy mixed-forest	Partially^2^
**Bais and yangas** 3	*E*, *G*	Attracted to bais and yangas for aquatic herbaceous food, minerals and water	Density negatively associated with increasing distance away from bais Density positively associated with increasing density of yangas	Yes
**Open roads**	*E,G,C*	Avoid open access roads with regular human activity	Density positively associated with increasing distance away from roads	Yes (*E,C*)No (*G*)
**Navigable rivers**	*E,G,C*	Avoid rivers with relatively regular human activity	Density positively associated with increasing distance away from rivers	No
**Human settlements**	*E,G,C*	Avoid human settlements	Density positively associated with increasing distance away from human settlements	No
**Logging history**	*E,G*	Attracted by the re-growth of herbaceous food plants in secondary forests following logging activities	Density positively associated with increasing time since start of logging activities (of first cycle of selective logging if more than one cycle)	Yes
	*C*	Deterred by loss of canopy cover and removal of fruiting trees by logging activities	Density negatively associated with increasing time since start of logging activities	Yes
**Distance to National Park**	*E,G,C*	Attracted to NNNP where human disturbance is low	Density negatively associated with increasing distance away from the NNNP border	Yes (*E,C*)No (*G*)
**Management plan status**	*E,G,C*	Do not avoid areas where negative impacts of human activities are mitigated	Density positively associated with higher conservation management status	Yes

1 Hypotheses and predictions are species-specific and not all covariates apply to all species: *E* = Elephant, *G* = Gorilla, *C* = Chimpanzee.

2 Not supported by model-based analysis but supported in part by design-based estimates by habitat type.

3 Natural forest clearings that provide a concentrated, year-round source of herbaceous food plants and minerals for several wildlife species. *Bais* are fed by a permanent running water source, whereas *yangas* are ‘closed’ with no surface water entry or exit point.

## Results

### Elephant and great ape abundance by management unit and habitat

Design-based distance sampling estimates of abundance of elephants and apes varied considerably between management units (**Tables 3** **&** **4**). We used a Z-statistic to compare densities between management units taking into account the dependence due to the common detection function [36].

**Table 3:** Elephant dung density (Dung piles/km^2^) and individual elephant density (Inds/km^2^) with 95% confidence intervals (95% CI) and percent coefficient of variation (%CV) for each survey stratum and for the landscape.

Survey stratum	L (km)	No. Dung piles	Dung piles/km^2^ [95% CI]	Inds/km^2^ [95% CI]	%CV^1^
NNNP	40.0	165	551.0 [407.3–745.3]	0.55 [0.40–0.75]	15.2
Kabo FMU	30.0	182	616.8 [405.4–938.6]	0.61 [0.40–0.94]	20.4
Pokola FMU	41.0	211	697.9 [406.8–1197.4]	0.70 [0.40–1.20]	26.7
Loundougou FMU	35.7	96	333.8 [161.5–689.8]	0.33 [0.16–0.69]	35.8
Mokabi	29.0	22	22.2 [7.1–69.6]	0.02 [0.007–0.06]	57.5
Bailly	48.0	161	432.4 [183.9–1016.7]	0.43 [0.18–1.0]	43.4
LTCR^2^	106.0	5	9.61 [3.3–28.3]	0.009 [0.003–0.03]	59.8
**Ndoki-Likouala Landscape**	**329.7**	**842**	**397.6 [298.3–529.9]**	**0.40 [0.29–0.53]**	**15.0**

Also shown is the total survey effort (L) and the total number of dung piles counted before truncation (No. Dung piles).

1 % CV calculated for individual density incorporates variance of dung decay and defecation rates.

2 Abundance estimate for LTCR was calculated by summing the abundance estimates from each habitat stratum. The density estimate for the whole LTCR is an average of the habitat-stratum specific densities weighted by stratum area. Log-based confidence intervals for abundance and density estimates were estimated from the components contributing to the variance for each habitat stratum using the delta method, and accounting for dependence due to the common detection function and sign creation and decay rates.

**Table 4:** Great ape nest density (Nests/km^2^) and individual density (Inds/km^2^), 95% confidence intervals (95% CI) and percent coefficient of variation (%CV) for each survey stratum and for the landscape.

Survey stratum	L (km)	Ape sp.	No. nests	Nests/km^2^ [95% CI]	Inds/km^2^ [95% CI]	%CV^1^
NNNP	40.0	*Apes*	*283*	*265.7 [174.0–405.7]*	*2.90 [1.90–4.44]*	*20.6*
		Gorilla	81	93.2 [53.9–161.3]	1.02 [0.59–1.77]	26.8
		Chimp	202	102.3 [61.7–169.8]	1.03 [0.61–1.71]	25.1
Kabo FMU	30.0	*Apes*	*175*	*232.2 [134.3–401.5]*	*2.54 [1.47–4.39]*	*26.2*
		Gorilla	119	197.6 [93.5–417.4]	2.16 [1.02–4.56]	36.1
		Chimp	56	39.3 [23.7–65.2]	0.39 [0.24–0.66]	24.5
Pokola FMU	41.0	*Apes*	*371*	*361.1 [214.9–606.7]*	*3.95 [2.35–6.64]*	*25.5*
		Gorilla	305	373.6 [207.4–672.8]	4.08 [2.27–7.36]	28.9
		Chimp	66	34.0 [14.2–81.5]	0.34 [0.14–0.82]	44.2
Loundougou FMU	35.7	*Apes*	*131*	*147.1 [89.2–242.4]*	*1.61 [0.97–2.65]*	*24.3*
		Gorilla	51	71.2 [31.3–161.9]	0.78 [0.34–1.77]	40.7
		Chimp	80	47.8 [25.6–89.4]	0.48 [0.25–0.90]	30.9
Mokabi	29.0	*Apes*	*15*	*20.8 [8.0–54.2]*	*0.23 [0.09–0.59]*	*47.0*
		Gorilla	8	14.1 [4.8–41.3]	0.15 [0.05–0.45]	53.5
		Chimp	7	5.2 [2.1–13.0]	0.05 [0.02–0.13]	45.5
Bailly	48.0	*Apes*	*157*	*131.7 [80.7–214.8]*	*1.44 [0.88–2.35]*	*24.2*
		Gorilla	75	78.8 [39.0–159.2]	0.86 [0.43–1.74]	35.1
		Chimp	82	36.6 [19.6–68.3]	0.37 [0.2–0.69]	31.2
LTCR^2^	106.0	*Apes*	*521*	*190.0 [126.5–285.3]*	*2.08 [1.38–3.12]*	*21.0*
		Gorilla	451	207.8 [152.1–283.9]	2.27 [1.66–3.11]	16.1
		Chimp	70	12.95 [6.6 –25.5]	0.13 [0.07–0.26]	35.9
**Ndoki-Likouala Landscape**	**329.7**	***Apes***	***1,653***	***197.9 [158.7–246.8]***	***2.16 [1.73–2.70]***	***11.3***
		**Gorilla**	**1,090**	**151.3 [113.5–201.9]**	**1.65 [1.24–2.21]**	**14.5**
		**Chimp**	**563**	**41.2 [31.4–54.0]**	**0.41 [0.31–0.55]**	**14.6**

Also shown is the total survey effort (L), the ape species (Ape sp.) and the total number of nests counted before truncation (No. nests).

1 % CV calculated for individual density incorporates variance of dung decay and defecation rates.

2 See Table 3 for methods used to estimate abundance, density and confidence intervals for the whole LTCR.

#### Elephants

Global elephant dung density for the Ndoki-Likouala Landscape was 397.6 dung piles/km^2^ (95% Confidence Intervals = 298.3–529.9) and elephant density for the landscape was estimated at 0.40 individuals/km^2^ (95% CI = 0.29–0.53; **Table 3**). The highest elephant density was found in the Pokola FMU, but there was no statistically significant difference at the 5% level of significance between density in this management unit and those recorded in the contiguous strata of the NNNP, Kabo FMU, Loundougou FMU and the Bailly. The lowest elephant densities were found in the LTCR and Mokabi, which were both significantly lower than densities recorded in NNNP and Kabo and showed a similar trend for Pokola, that approached but did not reach significance (LTCR: p = 0.062, Mokabi: p = 0.058).

**Figure 2:**
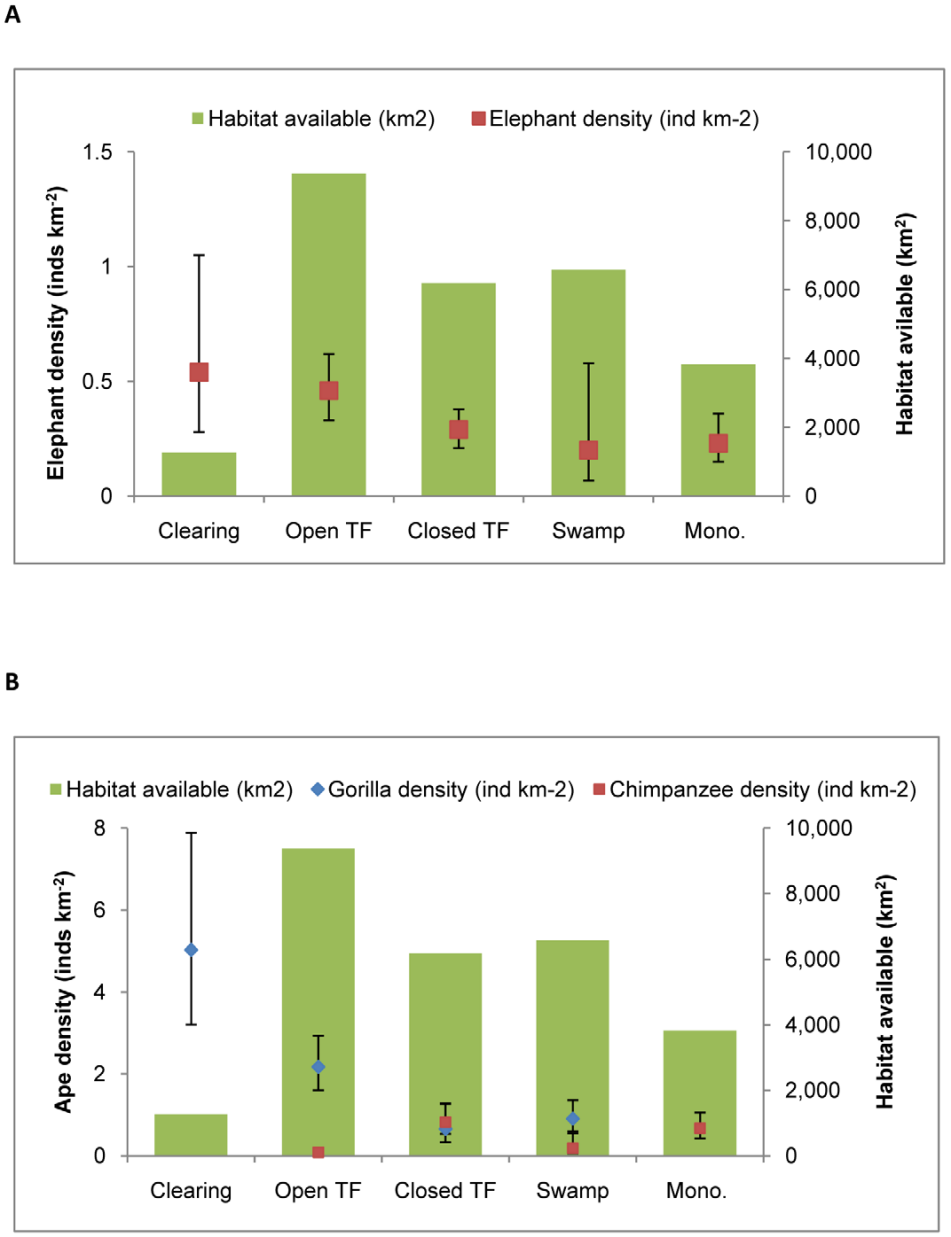
Elephant and ape density by habitat type. A – Elephant density, B – Great ape density; Clearing = natural forest clearings (*bais* and *yangas*) and light gaps, Swamp = swamp forest, Closed/Open TF = Closed-canopy or Open-canopy *terra firma* forest, Mono. = monodominant *Gilbertiodendron* forest.

Global estimates of elephant density by habitat type showed highest densities in forest clearings and closed-understory forest (**Figure 2**). Across survey strata, the availability of closed-understory *terra firma* forest (as measured on transects) was the sole and highly significant habitat predictor of elephant density (Elephant density = 0.00004_closed understory forest_−0.1159; R^2^ = 0.93; p = 0.0004). Overall elephant density was low in swamp forest.

#### Great apes

Global gorilla nest density for the Ndoki-Likouala Landscape was 151.3 nests/km^2^ (95% CI = 113.5–201.9) and gorilla density for the landscape was estimated at 1.65 individuals/km^2^ (95% CI = 1.24–2.21). Global chimpanzee nest density for the landscape was 41.2 nests/km^2^ (95% CI = 31.4–54.0), and chimpanzee density was estimated at 0.41 individuals/km^2^ (95% CI = 0.31–0.55) (**Table 4**).

Gorilla densities were higher than chimpanzee densities in all management units with the exception of the NNNP, where chimpanzee densities were highest (significantly higher than in all other management units in the landscape), estimated at 1.03 individuals/km^2^ (0.61–1.71), and comparable with gorilla density in this stratum (**Table 4**). Chimpanzee density in the Loundougou FMU was also high and significantly higher than all other management units with the exception of NNNP. Chimpanzee densities in Kabo, Pokola and the Bailly were comparable. Chimpanzee densities in Mokabi and LTCR were significantly lower than all other management units, with densities in Mokabi significantly lower than those recorded in the LTCR.

Gorilla density was highest in Pokola FMU with 4.08 individuals/km^2^ (2.27–7.36) and was significantly higher than all other strata in the landscape. Gorilla densities in Kabo and LTCR (swamp and *terra firma* forests) were also high, comparable with one another and significantly higher than all other management units with the exception of Pokola. Gorilla densities in the NNNP were significantly higher than those found in the Bailly and Loundougou. Gorilla densities in Mokabi were significantly lower than recorded in all other management units.

Estimates of gorilla density by habitat type showed highest nest densities in forest clearings and in closed-understory mixed forest (**Figure 2**). The availability of closed understory *terra firma* forest and forest clearings both showed a positive trend with gorilla nest density across management units. Availability of forest clearings was a weakly significant predictor of gorilla nest density at the stratum level (Gorilla nest density = 0.008626_Clearing_ + 0.049705, R^2^ = 0.58; p = 0.0454). Global estimates of chimpanzee density by habitat type showed highest nest densities in monodominant *Gilbertiodendron* forest and closed-canopy forest although neither of these two habitat types showed any clear trends with chimpanzee nest density across strata. Both gorilla and chimpanzee nest density in swamp forest were highly variable across different management units.

### Spatial models of elephant and great ape abundance at the landscape-scale

**Figure 3:**
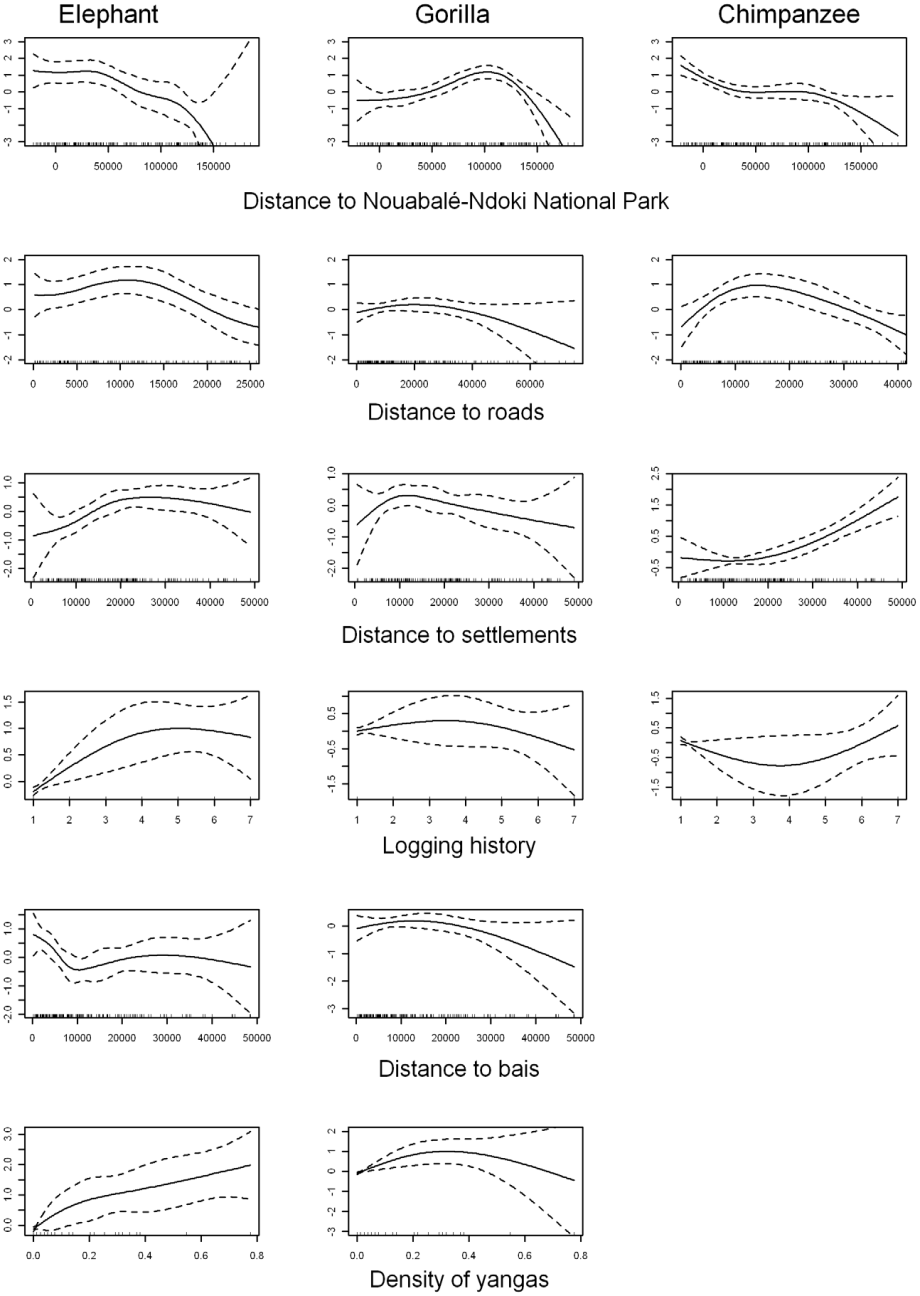
Estimated conditional dependence of sign densities on landscape covariates. Estimated conditional dependence of Elephant dung density (left column), Gorilla nest density (middle column), and Chimpanzee nest density (right column) on distance to the NNNP boundary (first row), distance to roads (second row), distance to settlements (third row), logging history (fourth row), distance to bais (fifth row), and density of yangas (sixth row). Estimates (solid lines) and confidence intervals (dashed lines), with a rug plot indicating the covariate values of observations (short vertical bars along each x-axis), are shown are shown. Y-axis scale can vary between species for a particular covariate.

Elephants, gorillas and chimpanzees responded differently to landscape-scale covariates (**Table 5**). Distance to the NNNP boundary explained 27%, 18% and 23% of the variance in elephant, chimpanzee and gorilla dung and nest counts, respectively. As hypothesised, both elephant and chimpanzee density decreased with increasing distance outside the NNNP boundary (**Figure 3**). Chimpanzee density decreased rapidly outside the NNNP up to a distance of 40km and increased rapidly inside its boundary. Elephant density within the NNNP boundary and at short distances up to about 20km outside its border was relatively stable, but then decreased rapidly with increasing distance away from the NNNP. In contrast to our hypothesis, gorilla density increased with increasing distance outside the NNNP boundary (**Figure 3**), up to distances of approximately 100km. Distance to open roads was an equally strong predictor of elephant and chimpanzee density, but had little effect on gorillas (**Table 5**). Both elephant and chimpanzee density increased with increasing distance away from roads, for distances up to 10km in the case of elephants, and 15–20km for chimpanzees (**Figure 3**).

**Table 5:** Results of the Generalized Additive Model analysis.

Covariate	UBRE Score (%)			Deviance Explained (%)		
	Elephant	Gorilla	Chimpanzee	Elephant	Gorilla	Chimpanzee
**Ecological**						
Vegetation	7.4349	12.228	6.506	10.70	2.22	5.39
Distance to bais	7.8486	12.175	-	6.57	2.52	-
Density of yangas	7.4299	11.813	-	10.80	5.21	-
**Human**						
Distance to roads	6.3223	12.215	5.6649	22.90	2.22	15.80
Distance to rivers	8.3302	12.440	6.8469	1.11	0.66	0.59
Distance to settlements	7.7767	12.131	5.6775	7.17	3.01	15.50
Logging history	7.4553	12.429	6.7482	10.40	0.63	1.86
**Management**						
Distance to NNNP boundary	5.9304	9.504	5.5046	27.00	22.5	17.80
Management status	7.7956	11.343	5.7975	6.78	8.70	14.00
Stratum	4.6769	9.826	4.9794	40.50	20.30	25.00
X coordinate	4.9942	10.545	6.0642	37.50	15.20	11.20
Y coordinate	5.4343	9.082	4.8911	32.80	26.1	26.50
**Combined model**	**1.7149**	**7.0473**	**4.0743**	**74.9**	**41.70**	**38.00**

The status of management planning (used here as a proxy measure for degree of threat mitigation) was a relatively weak predictor of chimpanzee density (14% of variance explained), and even less of a predictor of gorilla and elephant density (**Table 5**). Predicted density of all three species increased where management plans were either adopted or underway compared to where no formal management planning had taken place (Mokabi and Bailly). For elephants and chimpanzees, predicted density was significantly higher where management plans were adopted or where threat mitigation was most advanced (Kabo and NNNP) than where there was no management planning at all.

Distance to navigable rivers had very little predictive power for any of the three species. Distance to human settlements was a weak predictor of gorilla and elephant density, and only a moderately stronger predictor of chimpanzee density, accounting for 16% of the variance in the data for this species (**Table 5**). Both elephant and chimpanzee predicted density increased at greater distances from villages up to 40–50km for chimpanzees and approximately 25km for elephants, before density began to decrease (**Figure 3**). For both species, the effect was weak in very close proximity to villages. Logging history was a relatively weak predictor of great ape density, and a stronger predictor for elephants (10% of the variance explained). Due to the resolution of the landscape-scale dataset, logging history was modelled as a categorical variable (in 5 year blocks) rather than as a continuous variable, so short-term effects could have been missed. However, as hypothesized, species responded differently to logging history (**Figure 3**). The model predicted increased elephant and gorilla density with increasing time since logging up to a maximum of 15 years, before decreasing and eventually approaching values in unlogged forest. Even in areas subjected to 30 years of logging activity predicted elephant density remained higher than in unlogged forest. In contrast, the model predicted decreased chimpanzee density with increasing time since logging, again up to 15 years, after which it approached values in unlogged forest.

Vegetation type was a relatively weak predictor of density for all three species, particularly for apes, which may be attributed to the poor resolution of this covariate, and in particular the lack of discrimination between open and closed-canopy *terra-firma* forest. Natural permanent forest clearings (*bais* and *yangas*) were reasonably strong predictors of elephant density and to a lesser extent gorilla density. Predicted elephant density decreased with increasing distance from bais up to short distances of 5–10km (**Figure 3**). The response of gorilla abundance to bai proximity was less clear, staying relatively stable with distances up to approximately 20km before decreasing. Both elephant and gorilla abundance increased with higher densities of yangas (**Figure 3**).

Survey stratum had a considerable influence on the predicted distribution of all three species, accounting alone for 40%, 25% and 20% of the variance in elephant, chimpanzee and gorilla counts respectively (**Table 5**) and suggesting the influence of additional factors specific to individual management units that were not captured by our set of landscape covariates. Similarly, both X and Y coordinates were able to explain large amounts of the variability in the elephant, chimpanzee and gorilla count data and indicated north-south and west-east gradients in density across the landscape that were not fully explained by other covariates.

**Figure 4:**
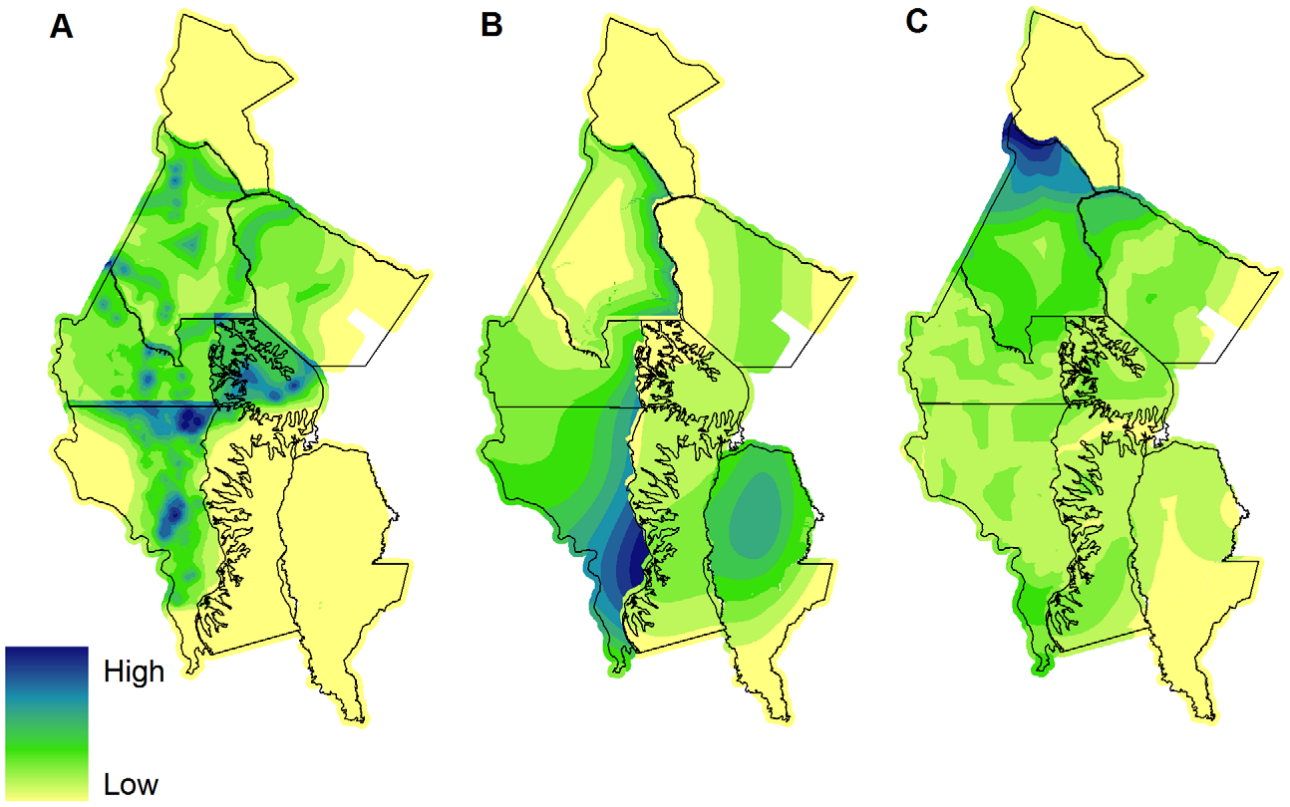
Predicted density surfaces from final composite models. A - Elephant dung density (Distance to bais, density of yangas, distance to roads, distance to NNNP boundary, stratum, Y coordinate), B - Gorilla nest density (Distance to NNNP boundary, stratum, X coordinate), and C - Chimpanzee nest density (Distance to NNNP boundary, distance to roads, stratum, Y coordinate). Density surfaces displayed in ArcGIS 9.2 (ESRI, Redlands, USA) using a Natural Breaks (Jenks) classification set to 10 classes.

In general our suite of landscape-scale covariates was a much better predictor of elephant abundance than of gorilla or chimpanzee abundance. A composite model for elephants with a low UBRE score and 75% of the variance in the data explained was selected that retained a total of six covariates (distance to bais, density of yangas, distance to roads, distance to NNNP, stratum and Y-coordinate; **Figure S1**). Composite models were also selected for gorillas (distance to NNNP, stratum and X-coordinate; **Figure S2**.) and chimpanzees (distance to NNNP, distance to roads, stratum and Y-coordinate; **Figure S3**), which explained 42% and 38% of the variance in the data respectively, and which were considered to provide the most biologically meaningful explanation of the data. Model-based abundance estimates for all three species were remarkably similar to design-based estimates (**Tables 6** **&** **7**), and composite models were used to generate density surfaces for all three species (**Figure 4**). Except for a moderate improvement in the precision of the landscape-wide abundance estimate of gorillas, and LTCR abundance estimates of chimpanzees and elephants model-based abundance estimation provided no notable gains in precision compared to the design-based results.

**Table 6:** Elephant design- and model-based abundance estimates (N and N′, respectively) with 95% confidence intervals (95% CI and 95% CI′) and percent coefficient of variation (%CV and %CV′) for each survey stratum and for the landscape.

Survey stratum	N [95% CI]	%CV	N′ [95% CI′]	%CV′^1^
NNNP	2,175 [1,595–2,966]	15.2	2,131 [447–3,309]	50.9
Kabo FMU	1,774 [1,160–2,713]	20.4	1,606 [217–2,869]	65.7
Pokola FMU	3,130 [1,817–5,392]	26.7	3,157 [508–5,590]	74.3
Loundougou FMU	1,406 [679–2,914]	35.8	1,230 [416–4,179]	66.5
Mokabi	59 [19–185]	57.5	95 [199–3,058]	70.0
Bailly	2,495 [1,059–5,880]	43.4	2,194 [672–4,891]	49.1
LTCR	37 [13–109]^2^	59.8^2^	39 [752–2,763]	43.9
**Ndoki-Likouala Landscape**	**11,076 [8,223–14,920]**	**15.0**	**10,452 [7,813–17,126]**	**22.5**

1 The model-based coefficient of variation %CV′ is directly comparable to the design-based %CV, as aside from the variation from encounter rate it also includes the variation from the detection function, dung production and dung decay. The model-based 95% CI′ only include variation due to variation in encounter rate.

2 See Table 3 for methods used to estimate abundance, density and confidence intervals for the whole LTCR.

**Table 7:** Great ape design- and model-based abundance estimates (N and N′, respectively) with 95% confidence intervals (95% CI and 95% CI′) and percent coefficient of variation (%CV and %CV′) for each survey stratum and for the landscape.

Survey stratum	Ape sp.	N [95% CI]	%CV	N′ [95% CI′]	%CV′^1^
NNNP	Gorilla	4,038 [2,331–6,994]	26.8	4,468 [2,746–12,939]	37.9
	Chimp	4,066 [2,434–6,795]	25.1	4,340 [385–2,906]	46.0
Kabo FMU	Gorilla	6,235 [2,950–13,178]	36.1	3,950 [1,499–10,921]	47.7
	Chimp	1,138 [682–1,899]	24.5	1,092 [231–2,197]	51.9
Pokola FMU	Gorilla	18,382 [10,198–33,132]	28.9	19,185 [2,991–14,848]	39.0
	Chimp	1,533 [637–3,691]	44.2	1,504 [506–3,537]	46.4
Loundougou FMU	Gorilla	3,292 [1,448–7,486]	40.7	2,876 [2,218–14,323]	43.0
	Chimp	2,028 [1,078–3,813]	30.9	1,830 [418–3,295]	76.4
Mokabi	Gorilla	411 [140–1,204]	53.5	424 [1,230–10,217]	47.3
	Chimp	138 [55–350]	45.5	160 [200–2,404]	53.5
Bailly	Gorilla	4,988 [2,466–10,086]	35.1	4,602 [3,961–18,284]	36.5
	Chimp	2,127 [1,134–3,989]	31.2	1,796 [723–4,161]	42.1
LTCR	Gorilla	8,919 [6,514–12,211]	16.1^2^	9,563 [4,016–10,546]	23.8
	Chimp	509 [257–1007]^2^	35.9^2^	623 [807–2,308]	27.1
**Ndoki-Likouala Landscape**	**Gorilla**	**46,264 [34,607–61,849]**	**14.5**	**45,068 [42,585–58,601]**	**9.0**
	**Chimp**	**11,541 [8,651–15,396]**	**14.6**	**11,345 [8,480–13,222]**	**15.2**

1 The model-based coefficient of variation %CV′ is directly comparable to the design-based %CV, as aside from the variation from encounter rate it also includes the variation from the detection function, nest production and nest decay. The model-based 95% CI′ only include variation due to variation in encounter rate.

2 See Table 3 for methods used to estimate abundance, density and confidence intervals for the whole LTCR.

## Discussion

Over recent years there have been increasing calls for an evidence-based approach to conservation investment and for reliable measures of success of different approaches to biodiversity management [37]–[40]. Our study in northern Congo provides the first evaluation of a landscape-scale conservation approach to conserving ecologically functional populations of elephants and great apes. We assessed the status of species in management units across different land-use types, and examined species response to varying human, ecological and management processes operating at the landscape-scale. We consider below the capacity of the survey design and estimation approaches employed here in providing a reliable evidence-base to support conservation decision making. Specifically we assess the extent to which the data improve our understanding of (a) the spatial processes governing the distribution of great apes and elephants at the landscape scale (**Table 2**), and, (b) the effectiveness of different management strategies in conserving ape and elephant populations, and suggest design improvements for long-term monitoring programs.

### Land-use strategies and conservation management

There was considerable variation in abundance between species across the landscape. Land-use type - protection, logging concession and community-based natural resource management - itself had no consistent effect on the abundance of different species. However, the degree of wildlife management intervention within different land-use types had an overwhelming effect on species abundance: elephant and gorilla populations in certain managed logging concessions were comparable with, and in the case of gorillas higher than, density estimates in the NNNP; indeed gorilla density estimates in the Pokola logging concession are some of the highest gorilla densities recorded in Central Africa (reviewed in [41]). In contrast, in the Mokabi concession, both logging and hunting intensity were high, wildlife management absent, and the abundance of all three species was consistently lower than all other management units (with the exception of elephants in LTCR). In the absence of any formal anti-poaching activities, Mokabi is subject to considerable and uncontrolled hunting pressure from across the Central African Republic border: the frequency of spent gun cartridges found during this study was over 18 times higher than the mean value from all other management units. This difference in species abundance between managed and un-managed logging concessions is particularly striking at a time of accelerated expansion of logging activities and associated socio-economic change across the whole landscape; between 2000–2006 the population of the five principal logging towns in FMUs under the principal logging concession holder Congolaise Industrielle des Bois (CIB), including Pokola, (**Table 1**) grew by 69% [42]. Our surveys also represent the first wildlife assessment of CIB's Kabo FMU since it was granted Forest Stewardship Council (FSC) certification for good environmental, social and logging practices in May 2006, with FSC certified status subsequently extended to the Pokola FMU in May 2008 [43].

Extremely low elephant densities in the LTCR, even after accounting for the low probability of detection of dung piles in swamp forest, are a legacy of commercial elephant hunting dating from the 1970s and 1980s, rather than a reflection of relatively recent community-based management efforts. Low elephant densities were already being reported from this region in the late 1980's [44].

### Ecological factors

Ensuring that wildlife populations maintain access to ecologically important resources that lie outside of strictly protected areas is a fundamental element of management planning at the landscape scale and a primary objective of our monitoring program. Large areas of important habitat for gorillas and elephants lie outside the strict protection of the NNNP in the form of dense herbaceous forest undergrowth, [45]–[48], swamp forests [49]–[51], and natural forest clearings that are rich in minerals [48], [52], [53]. In frontier forests such as parts of northern Congo, where remote and inaccessible areas are fast being penetrated by commercial logging, the key to successful intervention is to identify and protect critical habitats and corridors before they are degraded or irreversibly impacted by poaching [1], [54]. Our results indicate that measures have been broadly successful in maintaining access by elephants and gorillas to key resources such as *bais* and *yangas*. Vegetation type was not identified as a major ecological covariate for any species in our landscape model. We suggest this is partly due to the coarse resolution of the spatial vegetation covariate dataset and specifically its failure to discriminate between closed and open-canopy *terra firma* forest; an important factor in determining local abundance of western gorillas and chimpanzees respectively [41], [46]. This is also supported by results from our design-based estimation of density by habitat-type: availability of open canopy/closed understory forest was a strong predictor of elephant density and weaker predictor of gorilla density respectively indicating that these factors were important in explaining distribution. Logging, by directly altering forest structure, can further confound the effects of habitat preferences on species abundance, both in time and in space. Recently logged forests (<15 years) had a positive impact on elephant density and, to a lesser extent gorillas, supporting the prediction that in the absence of poaching forest elephants can occur in high densities in logged forest due to an abundance of preferred herbaceous food plants [45]. Conversely, chimpanzee density was highest in the two management units with the largest total area of mature closed-canopy *terra firma* forests and the shortest history of logging (the NNNP and Loundougou FMU): with these two strata accounting for over 50% of the total chimpanzee population.

### Human impacts

For elephants, proximity to roads and the NNNP boundary remained a stronger predictor of distribution than any ecological variable considered here. We found four poached elephant carcasses across the landscape during our survey, all of which were adjacent to logging roads in each of Mokabi, Pokola, Kabo and Toukoulaka FMUs, indicating that poachers are profiting from the road networks to penetrate deeper into the forest away from urban settlements to hunt elephants, even in the CIB concessions (Pokola, Kabo, Toukoulaka and Loundougou) where considerable efforts have been taken to reduce poaching and trafficking of bushmeat and other wildlife products on logging roads [32]. For elephants, the NNNP, as the only strictly protected area with no permanent human habitation or roads and no sign of poaching during our survey, clearly provides a critical and very necessary element of the conservation landscape, with density declining rapidly at more than 50km beyond its border. Chimpanzee density also increased with increasing distance from roads and from villages, whereas gorilla density showed little response to either roads or settlements (*cf*. [48]). With the exception of Mokabi, it appears that apes are not targeted specifically for commercial hunting (*cf*. [55], [56]), although opportunistic hunting does occur [42]. The response of chimpanzees to villages and roads may be indicative of a general response to increased human disturbance [57] or to habitat modification. In general, our results support observations elsewhere that chimpanzees appear to be more sensitive than gorillas to logging and human disturbance outside of protected areas [46], [58]. The fact that gorilla abundance was found to increase with increasing distance away from the NNNP boundary lends additional weight to this argument, with two caveats: firstly, the model response to this covariate is likely confounded by very high densities in the swamp forests of LTCR to the south, and secondly, gorilla density in logged forest with no formal anti-poaching measures in place (Mokabi) was very low.

### Recommendations for management-based monitoring programs

We emphasize the importance of rigorous stratified design-based monitoring programs for assessing abundance under varying degrees of management intervention and human impact. At the level of individual management units, our survey design succeeded in capturing the spatial heterogeneity in abundance of all three species. Furthermore, we stratified our estimates at the same scale at which management plans are adopted and ultimately evaluated and therefore the scale at which our monitoring program stands to have the greatest impact on policy decisions, including FSC certification of logging concessions. Estimates were obtained with adequate, pre-defined levels of precision (25% CV for elephants and apes), with some notable exceptions: small sample sizes (LTCR elephants), lower-than expected encounter rates (Mokabi) and highly clumped distributions within some survey strata (e.g. elephants in Bailly and Loundougou, **Figure 4**). Modifications to the sampling design such as additional sub-stratification and re-allocation of survey effort would improve the precision of estimates and facilitate future monitoring work.

We also emphasize the importance of developing appropriate field protocols to distinguish between the two ape species during nest surveys [59], which has been overlooked in the literature, particularly in evaluating the response of sympatric apes to logging activities in Central Africa. An additional source of bias in density estimates from counts of indirect sign (nests and dung) is introduced with the use of conversion factors: production and decay rates [60]–[64]. In reality, the process of production and decay of both nests and dung is the result of a complex interaction of multiple factors and will likely vary both temporally and spatially across the landscape. We acknowledge that our use of fixed decay and production rates does not substitute for survey-specific and spatially representative estimates of these processes but, rather, serves as a foundation for future improvement. At a minimum, habitat-specific estimates of decay rates are needed and, at large spatial scales, the use of retrospective, or ‘two-visit’ decay rate estimation methods [65], [66] may be the most appropriate and cost-effective approach. Of additional relevance to nest-count surveys is the role of different land-use practices and levels of human disturbance on ape nesting behaviour and potential re-use of nests [61].

Model-based approaches are useful in examining the responses of species to different landscape processes and can be a powerful tool in influencing management and policy [67], [68]. In order to make sense of the inherent complexity of the landscape system, we used a spatially-explicit hypothesis-driven approach to evaluate assumptions of species response to threats, environmental factors and management interventions. In addition, model-based abundance estimation has the potential to improve precision by explaining more of the variation in the survey data through the use of covariates. However, confounded variables and missing covariates, manifested here by the relatively important effects of stratum and X/Y coordinates, combined with the low resolution of certain datasets, were a limiting factor in improving precision for any of the species estimates and in biologically-based interpretation of models at this spatial scale, particularly for great apes. For example, a finer resolution habitat classification map for the entire landscape would have been useful, as well as data on the availability of fruiting trees: elephants in the Ndoki landscape have been known to migrate across management unit borders according to rainfall regimes and fruiting patterns [69], [70]. However, given the resolution of our transect data we were only able to provide very coarse-scale interpretation of landscape-scale processes. For wide ranging species, such as elephants, these spatial scales are appropriate to the ecology of the species. However, for gorillas and chimpanzees there remains considerable uncertainty in our models. The response of great apes to local variation in environmental conditions, different logging practices and associated changes in forest structure and disturbance typically occur at smaller spatial scales [46], [49], [71]. We therefore recommend that landscape-scale monitoring programs for apes and elephants rely fundamentally on periodic implementation of design-based surveys to estimate abundance in management units relative to management objectives, but that these are complemented by small-scale targeted monitoring or research programs that evaluate the response to specific management actions of different variables (e.g. ape abundance, nesting behaviour, habitat use), using controlled or experimental designs that can disentangle the effects of human, management and ecological factors over time and inform management practice accordingly. Finally, we strongly recommend that any long-term biological monitoring program is interspersed with punctual assessment of threats including poaching and diseases such as Ebola, through standardized surveillance systems that can function as early warning signals of rapid population decline and facilitate short-term management intervention.

The results of this study represent data from a single snapshot in time, and species response to both the human, management and ecological processes examined here, are likely to vary in time as well as space. Whilst negative human impacts such as poaching can have rapid and drastic consequences on wildlife populations there is likely to be a time-lag between targeted conservation interventions and population response or recovery, and for long-lived and slowly reproducing species such as apes and elephants this requires long-term monitoring which is typically outside the time frame – or budgets - of most conservation projects. To ensure sustainability, the responsibility for biodiversity monitoring needs to be institutionalized amongst the landscape management agencies. Commercial logging concessions are typically allocated for 30-year leases in Congo, and forestry management plans that address wildlife management are now required by Congolese law. The formal incorporation of scientifically-rigorous monitoring guidelines into forestry management plans, and into the criteria of FSC and other timber certification schemes, is an important step in evaluating the benefits of these policy measures for wildlife. This in turn provides an opportunity for scientists and conservation practitioners to engage with the private sector in order to significantly improve the conservation outlook for elephants and great apes.

## Materials and Methods

### Ethics statement

All research was conducted using observation of indirect signs of animals (dung and nests). Permission for the research was granted under a Memorandum of Understanding between the Wildlife Conservation Society and the Government of the Republic of Congo.

### Study area

The Ndoki-Likouala Conservation Landscape extends across 27,970 km^2^ of contiguous lowland forest in northern Congo, from the Sangha River in the west through typical Guineo-Congolian lowland rainforest (*sensu* [72]) towards swamp forest in the east (**Figure 1**). The terrain is relatively flat and altitude varies between 300–600m. Climate is typically bimodal, with a pronounced drier season between December and March and a long rainy season between August and November, with a short wet and dry period between April–May and June–July respectively. The landscape is renowned for its intact assemblages of large forest mammals, including western gorillas, chimpanzees, forest elephants, forest buffalo *Syncerus caffer nanus* and bongo *Tragelaphus eurycerus*.

Human population density in the landscape is low (∼1.5 inhabitants/km^2^, **Table 1**), with the largest population centres clustered around logging towns (**Figure 1**). Pokola is the largest settlement in the landscape (∼13,417 inhabitants), and is the headquarters of the principal logging concession holder, CIB (Congolaise Industrielle des Bois). Logging activities dictate road access in the landscape – all roads are private logging roads, with the exception of the public road linking Epéna in LTCR to the Likouala provincial capital Impfondo east of the landscape (**Figure 1**). All management units, with the exception of Mokabi (a commercial logging concession) and the Bailly and Bodingo swamps (hereafter Bailly) were undergoing wildlife conservation interventions at the time of the surveys.

### Survey design

Line transect distance sampling [36] was used to estimate densities of elephants, gorillas and chimpanzees from counts of elephant dung piles and great ape sleeping nests respectively [63], [73]. Distance sampling approaches explicitly allow for the estimation of detection probability during analysis, and thus account for a major potential source of bias in density estimation [10]. Estimates of production and decay rates of both elephant dung and great ape nests were used to convert sign density into animal density [36]. The survey was designed and the results analyzed using the Distance 5.0 (release 2) software [74].

**Figure 5:**
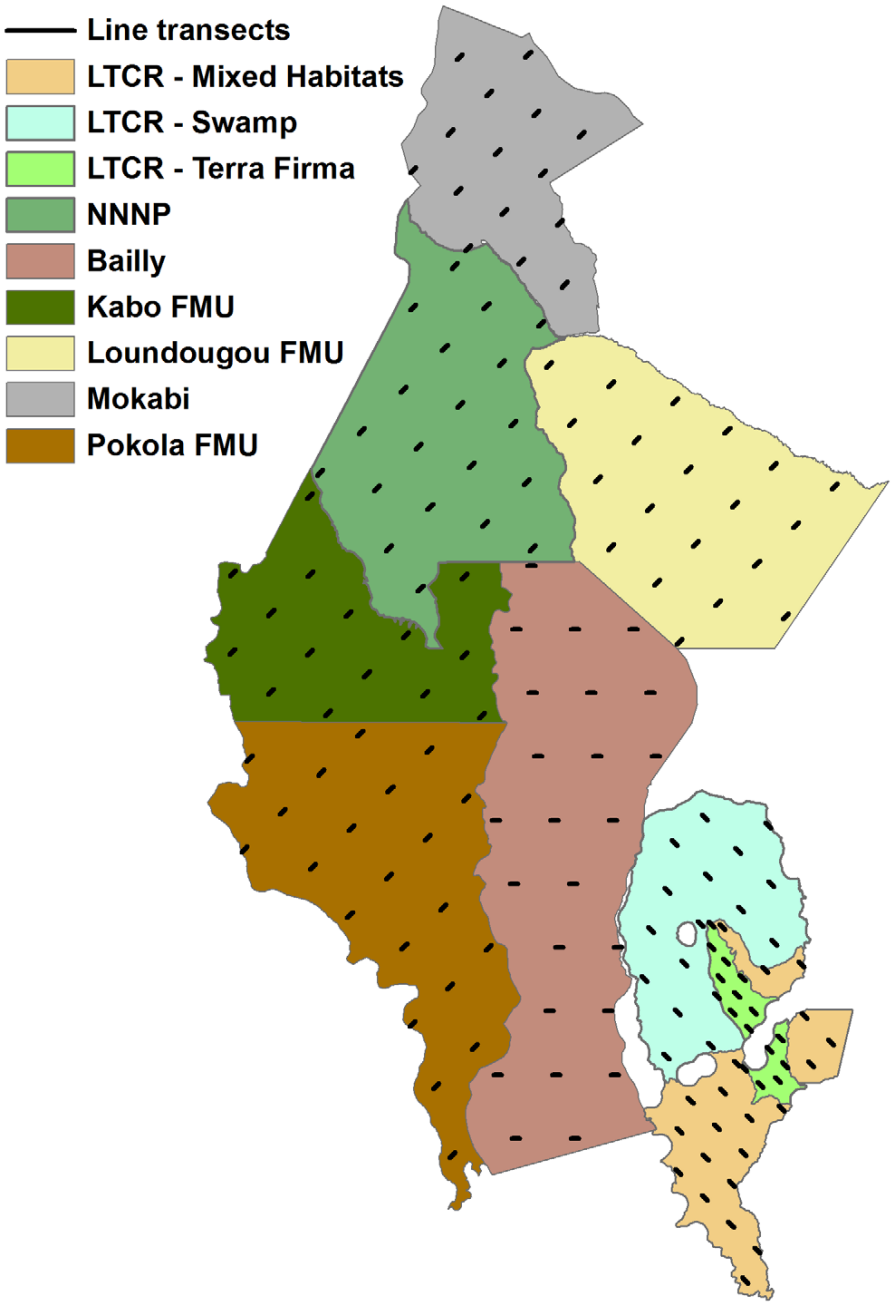
Landscape survey strata and transect placement. NNNP = Nouabalé-Ndoki National Park, LTCR = Lac Télé Community Reserve, FMU = Forestry Management Unit.

The landscape was stratified according to individual management units – Forestry Management Units (FMUs), Lac Télé Community Reserve (LTCR), Nouabalé-Ndoki National Park (NNNP) and unclassified swamp forest. Survey stratum limits followed the official limits in government or land-use decrees, with three exceptions. Firstly, surveys in the Mokabi-Dzanga FMU was restricted to the sector south of the main road between Congo and Central African Republic, and subsequently referred to as ‘Mokabi’ in this paper. Previous surveys had found the area north of this road to be mostly denuded of large mammal populations [19], and it does not therefore constitute an immediate monitoring priority. Secondly, placement of transects using the Distance software within the Toukoulaka FMU proved difficult because of its convoluted boundary between the *terra firma* forests to the west and the swamp forests to the east (**Figure 1**). To facilitate the survey design process, this area was therefore combined with the neighbouring Bailly swamps. Finally, the LTCR was sub-divided into three strata according to broad habitat type; swamp forests, mixed *terra firma* forests and ‘mixed’ forest type (including seasonally flooded forests, riparian forests and savannah), as forest type has been shown to strongly influence gorilla abundance in this protected area [49]. In total, nine different survey strata were defined (**Figure 5**). A 3km buffer zone around each of the villages in the swamp forests was excluded from the survey zone. Swamp forest presents a natural barrier to human access and we wished to reduce the risk of transects being used by hunters to penetrate deeper into the forest.

Using the Distance software line transects were placed systematically with a random start within each survey stratum, using the “Systematic Segmented Trackline Sampling” design class within the automated design component of the software. Sampling effort represented a balance between obtaining sufficient estimator precision on one hand, and the financial and logistical constraints of conducting surveys across such a large and relatively inaccessible landscape on the other hand. In redressing this balance, the sampling plan for this monitoring program aimed for a maximum coefficient of variation (CV) of 25% for stratum-specific density estimations of elephants and great apes [19]. The sampling design also assumed at least five repetitions in order to detect a minimum 30% change in population size over time with 75% statistical power to detect a true trend, and 10% probability of falsely detecting a trend when the population is stable (TRENDS: [75]). In calculating the total sampling effort required in each stratum for a target CV of 25% [36] we used encounter rates of elephant dung and great ape nests in each of the survey strata from prior baseline data collected independently in each of the management units [19], [48], [49].

All transects were 2km in length and the number of transects within each stratum varied between 15 and 24, with a total of 168 transects placed across the landscape. To improve precision in abundance estimates, transects were placed perpendicular to the main water courses so that these transects run approximately parallel to vegetation gradients and potentially associated gradients in wildlife density (**Figure 5**).

### Data collection

The survey was conducted between February and August 2006 during the dry (or low-water) season. A total of 10 field teams were deployed simultaneously across the survey zone, with two to three teams surveying a single stratum at any one time. Individual strata were surveyed to completion in the shortest time possible before continuing to the next, in order to minimise any seasonal variation in abundance within a single stratum. Due to extreme flooding two transects were not completed, resulting in a revised survey effort of 329.7km across 166 transects.

Field teams were composed of eight or nine members, including a principal observer responsible for observations of elephant dung and other signs on the ground and a second observer responsible for nest sightings in trees. Permanent transects were not cut but rather a straight line bearing was followed through the forest with minimal impact using only secateurs where possible to open the transect sufficiently to enable passage. A GPS point was taken at the beginning, mid-point and end of each transect. All changes in forest type along the transect were noted. All observations of elephant dung and elephants paths, great ape nests and dung and all signs of human activity, together with associated forest type, age of sign and hip chain distance along the transect were recorded. For elephant dung and great ape nests the perpendicular distance between the transect and the centre of the sign was recorded.

#### Forest type

A total of 22 different forest or habitat types were recorded during the survey. For the analysis, these were pooled into six distinct categories that were considered to be ecologically relevant to elephants and great apes, and for which sufficient observations were available: 1) monodominant *Gilbertiodendron* forest, 2) mixed-species *terra firma* forest with open-understory, 3) mixed-species *terra firma* forest with closed understory, 4) swamp or flooded forest, 5) forest clearings including natural permanent clearings (known as ‘*bais*’ and ‘*yangas*’) and ephemeral clearings or light gaps, and 6) other habitat types (roads, rivers, savannahs) (see also **Figure 1**).

#### Great apes

Construction type for all great ape nests [76], and for tree nests the height and species of tree in which the nest was built, were recorded. Nest groups were defined as all nests created by the same ape species and of the same age class created less than 50m from each other. Each nest was classified as definitely gorilla or chimpanzee if verifying signs (faeces, odour or hair) were present. Chimpanzees have not been recorded building regular ground nests in this region and so all nests on the ground were attributed to gorillas and any associated arboreal nests of the same age class as the nests on the ground also to gorillas [77]. For the remaining nests in trees the methodology outlined in [76] was followed: for those tree nests where the nest building ape species could not be verified by associated signs the species was recorded as ‘ape’. Nest age and construction definitions were based upon [77].

#### Elephants

During data collection the elephant dung piles were classified into five classes (A, B, C1, C2, D, E) based on their state of decomposition and using the system developed by [78]. Dung piles categorized as age class E are considered decomposed and were removed during analysis.

### Data analysis

#### Attributing nests to ape species

A total of 1,653 ape nests were observed, of which 918 nests were directly attributed to gorillas, 219 nests to chimpanzees and 516 nests (31%) to unknown ‘ape’ species. In order to correctly attribute these 516 ‘ape’ nests to either gorillas or chimpanzees, we applied a logistic regression model to a set of explanatory variables associated with 1,137 known gorilla and chimpanzee nests [59]. We used the statistical software R Ver. 2.8.1 [79] to construct a series of Generalized Linear Models (GLMs) using a binomial function with a logit link. A variety of models were constructed using different combinations of six predictor or discriminatory variables: survey stratum, habitat type (mixed species *terra firma* forest; monodominant *Gilbertiodendron* forest; Marantaceae forest; swamp forest; *Raphia* swamp – a specific sub-category of swamp forest; and logged forest), nest height, forest understory (a binomial variable indicating either closed or open), nest type (also a binomial variable indicating either tree or ground nests) and tree species (a potentially important yet problematic variable due to a large number of missing values). We compared the residual deviance of each model to the residual deviance of the corresponding null model, using Pearson's 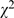 statistic. For each model, the probability of nest membership to either chimpanzees or gorillas was calculated as the response variable for each nest. Where nests were differentially assigned to different species within a single nest group, we calculated a mean probability value for the nest group and then manually re-assigned individual nests to the species indicated by that probability. Criteria for model selection was based upon the proportion of nests with known builders that were correctly assigned, as well as the Akaike's Information Criterion (AIC) value [80]. The final model retained a total of four important discriminatory variables (habitat type, nest height, forest understory and nest type) which succeeded in accurately assigning a total of 91% of nests with known builder as either chimpanzee or gorilla.

#### Standard distance sampling density and abundance estimates

Distance 5.0 (release 2) software [74] was used to estimate encounter rate, detectability, density and abundance of elephants, gorillas and chimpanzees. If all elephant dung piles or great ape sleeping nests – hereafter collectively referred to as indirect signs - located on the line were detected with certainty, then the density of any of the three types of indirect signs in the study area surveyed (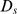) is estimated as:
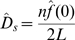
where 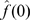 is the probability density function of the perpendicular distances evaluated at zero distance and 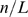 is the encounter rate. The density of animals (elephants, gorillas or chimpanzees) 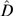 is obtained by dividing the estimated density of indirect signs 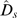 by the estimated sign production rate and average time to decay [36]. The density of animals is multiplied by the surface area 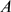 of the study area to obtain the corresponding abundance estimate 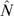. The methods for estimating these parameters, as well as the asymmetric log-based 95% confidence intervals for density and abundance are described in [36]. For great apes, individual nests were considered for analysis as opposed to nest groups. This can potentially underestimate variance in the density estimate, but at the same time avoids the issue of inaccurately estimating nest group size. Moreover, both the nest decay rates and logistic regression model for species discrimination are calculated at the level of individual nests rather than nest groups.

Two separate design-based analyses were completed for each of elephants, gorillas and chimpanzees. For the first analysis stratified estimates for encounter rates, density and abundance were obtained for each survey stratum and the data were pooled to estimate detectability (models that stratified detectability by survey stratum were also considered). Finally a global estimate of density and abundance was obtained for the entire landscape. Given the heterogeneity of habitat types in the Ndoki-Likouala, in the second analysis we stratified both encounter rate and detectability by habitat type. Density and abundance estimates were obtained for each habitat stratum and also globally. We were not able to provide density estimates for all habitats as a result of too few observations in certain classes (clearings for chimpanzees, *Gilbertiodendron* forest for gorillas, and roads/savannas/rivers for all of elephants, gorillas and chimpanzees). In spite of this, the global abundance estimates for the first and second analyses were similar for gorillas, chimpanzees and elephants.

The variance of encounter rate for each survey stratum and also for each habitat type within each stratum used in the first and second analysis respectively, was estimated empirically taking each transect line as a sampling unit. To improve model fit data were right truncated and grouped into distance intervals. For the detection function AIC was used in model selection and the results of the 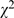 goodness-of-fit test were also considered.

#### Production and decay rates for ape nests and elephant dung piles

Due to the logistical constraints imposed by such a large survey landscape we did not obtain landscape-wide estimates of dung defecation and decay rates for elephants or nest creation and decay rates for great apes during this survey. Instead we used existing data from other studies conducted at specific sites within our landscape or from landscapes within the same biome, in order to convert sign densities into estimates of ape and elephant density. We present both sign density estimates (obtained from this survey) and individual density and abundance estimates (using these published conversion factors) in order to distinguish between these two processes.

For gorillas and chimpanzees, we used a nest decay time of 91.5 days (SE = 1.67) for both species obtained from extensive line transect surveys conducted in the NNNP [41]. We used a nest production rate of 1.09/day (SE = 0.05) for chimpanzees obtained from studies of habituated groups conducted in the NNNP [41]. We assumed a nest production rate of one per day for gorillas in Gabon [76] (no published standard error value available). For elephants, we used a dung decay time of 51.3 days (SE = 2.81), estimated from a three-year study also conducted in the NNNP [81] and a dung defecation rate of 19.76 dung piles/day (SE = 0.23) obtained by [82] for forest elephants in Cameroon.

For dung and nest decay rates, we ensured that the criteria used to define ‘disappearance’ in our survey were the same as those used in the original sign decay study. Furthermore, standard errors of decay and production rates used in our analyses were combined with estimated standard errors of sign encounter rate on transects and variability associated with detectability, and incorporated in the overall estimate of variation of the abundance estimates [36], [63].

#### Landscape spatial models

Generalized Additive Models (GAMs) were used to evaluate predictions of the distribution of apes and elephants (**Figure 3**). GAMS are particularly suitable for the interpretation of ecological data given their flexibility and capacity for non-linear responses that potentially mirrors how animals respond to fluctuations in their environment. The best composite models with multiple covariates were also used to produce density surfaces (**Figure 4**) and estimate abundance (per management unit and globally) for each of the three species (**Tables 6** **and** **7**).

Environmental, human, and management spatial covariates were considered, and a series of *a priori* hypotheses [80] about the distribution of apes and elephants were formulated on the basis of our knowledge of the ecology and behaviour of these species (**Table 2**; [19], [41], [49]). Human-activity, or potential threat variables included both distance-based proxies for hunting access [19], [83] and logging history [48]. Management variables were largely at the scale of individual land management units, and incorporated macro-level land-use planning. The choice of covariates was scale-dependent and thus limited to those covariates that varied at the landscape-scale and at similar resolution to our transect placement. A total of nine covariates were retained for the final analysis (**Table 8**). We also included both survey stratum and X/Y coordinates as covariates to account for possible geographical gradients in abundance or stratum-level effects that were not captured by our other landscape-scale covariates. Covariate values for analysis were obtained at the midpoint of each 2km transect (**Table 8**).

**Table 8:** Covariates used for Generalized Additive Modelling analysis.

Covariate	Sp.^1^	Method of Calculation	Values^2^	Method of data capture (and source)^3^
Vegetation type	*E,G,C*	Majority vegetation type within 1km circle radius	*Gilbertiodendron* forest; mixed forest; Savannah; Agriculture; Swamp; *Raphia* swamp; Earth/Roads; Water; Marantaceae forest	9-class land-cover reclassification of a partial coverage 18-class vegetation grid derived from Landsat 7 ETM+ imagery (WCS Congo/Woods Hole Research Center, USA)
Distance to bais	*E,G*	Euclidean distance (km) to all bais	13.2(±0.9)	Digitized from 1∶200,000 Topographic map and Landsat 7 ETM+ satellite imagery (WCS-Congo)
Density of yangas	*E,G*	Density of yangas within a 5km moving window	0.03(±0.008)	See ‘DISTBAIS’
Distance to roads	*E,G,C*	Euclidean distance to all roads accessible by vehicles at time of and in year preceding surveys	18.2(±1.3)	GPS data for logging roads in Kabo, Pokola, Loundougou & Toukoulaka (CIB-Pokola); digitized from 1∶1million map (WCS-Congo)
Distance to rivers	*E,G,C*	Euclidean distance to all rivers navigable by canoes	14.7(±0.8)	Digitized from 1∶200,000 map (WCS Congo)
Distance to settlements	*E,G,C*	Euclidean distance to permanent towns, villages and camps	18.5(±0.9)	GPS data for camps/logging towns; digitized from 1∶200,000 and 1∶1 mil. maps (WCS Congo)
Logging history	*E,G,C*	Number of years since start of commercial logging operations	Unlogged; <5 years; 5–10 yrs; 10–15 yrs; 15–20 yrs; 25–30 yrs; >30 yrs	Spatial limits defined by VMA (Maximum Annual Wood Volume) (CIB-Pokola/WCS Congo archives)
Distance to NNNP boundary	*E,G,C*	Euclidean distance from NNNP boundary^4^	64.3(54.0)	Spatial limits defined in legal decree (CNIAF, National Monitoring and Inventory Agency, Congo)
Management status	*E,G,C*	Status of formal management planning per unit	Underway; Adopted; None	WCS Congo Project archives

1 E = Elephant, G = Gorilla, C = Chimpanzee.

2 Mean values (with standard errors) shown for continuous variables (grid cell values corresponding to 2km transect mid-point); categories shown for factor variables.

3 We provide the original method of data capture, where known. For some covariates, data were collated from different sources to ensure landscape-wide coverage.

4 Negative distance values inside the NNNP boundary.

We fit a series of GAMs to sign count data (nests or dung) from the landscape surveys of the form:
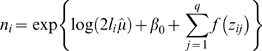
where 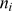 denotes the number of signs detected on the *i* ^th^ transect, 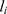 the length of the *i* ^th^ transect and 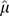 is a site-specific estimate of the effective strip half-width calculated using the Distance 5 software. The term 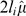 gives the area effectively surveyed on transect *i*. 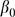 is the intercept, and 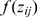 is a smooth function of the *j* ^th^ covariate 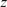 associated with the *i* ^th^ transect. By including area effectively surveyed as an offset term in the model, sign density is in effect being modelled. The models were fit in R [79] using the *mgcv* package [84]. Forward model selection was based on the Un-Biased Risk Estimator (UBRE) criterion and the percent deviance explained was also considered [84]. In addition, the standard diagnostic plots (Normal Q-Q, residuals vs. linear predictor, histogram of residuals, response vs. fitted values) were used in model selection and assessment of fit. Cubic regression splines were used to fit the smooth functions and to avoid over-fitting the degrees of freedom were restricted in the final models for all the covariates, and these assumed a Poisson distribution and log link. Covariate grids were created for the landscape survey area at 250m resolution and predicted density surfaces for signs were generated for the entire landscape from selected composite models. Estimates of chimpanzee, gorilla and elephant abundance from the fitted model were obtained by applying the same conversion factors previously described for the production and decay of signs. To estimate variance and percentile confidence intervals nonparametric bootstrapping was used [85]. A total of 999 bootstraps were conducted for each species during which replicate transect lines, assumed to be independently and identically distributed, were resampled at random and with replacement until each bootstrap resample was the same size as the original number of transects. Abundance estimates were obtained from the resampled data conditioning on the original model fit. The estimates were ordered from smallest to largest and the 25^th^ and 975^th^ value was used to define the percentile confidence interval. To obtain the total variance of the abundance estimate (expressed as a coefficient of variation) the sample variance of the abundance estimates from the resampled data predictions was combined with the variance associated with the detection probability and the production or decay rates using the delta method [86].

### Public access to data

All data currently reside in the public domain. Raw transect data on great apes has been uploaded into the A.P.E.S database (http://apes.eva.mpg.de/). Raw transect data on elephants has been uploaded into the IUCN African Elephant Specialist Group's African Elephant Database (http://www.african-elephant.org/aed/index.html). The data can be accessed from these sites by interested third parties through formal requests.

## Supporting Information

Figure S1 The composite model for elephant dung density. Estimated conditional dependence of elephant dung density on distance to bais, density of yangas, distance to roads and distance to the NNNP boundary. Estimates (solid lines) and confidence intervals (dashed lines), with a rug plot indicating the covariate values of observations (short vertical bars along each x-axis), are shown. Stratum and Y coordinate were also included as covariates. Note that y-axis scale is selected optimally for each covariate.
(0.10 MB TIF)

Figure S2 The composite model for gorilla nest density. Estimated conditional dependence of gorilla nest density on distance to NNNP boundary. Estimates (solid lines) and confidence intervals (dashed lines), with a rug plot indicating the covariate values of observations (short vertical bars along each x-axis), are shown. Stratum and X coordinate were also included as covariates. Note that all plots have the same y-axis scale.
(0.03 MB TIF)

Figure S3 The composite model for chimpanzee nest density. Estimated conditional dependence of chimpanzee nest density on distance to roads and to the NNNP boundary. Estimates (solid lines) and confidence intervals (dashed lines), with a rug plot indicating the covariate values of observations (short vertical bars along each x-axis), are shown. Stratum and Y coordinate were also included as covariates. Note that all plots have the same y-axis scale.
(0.05 MB TIF)
