# Supplementary figures and images for: Monitoring Great Ape and Elephant Abundance at Large Spatial Scales: Measuring Effectiveness of a Conservation Landscape
Source: PLoS One. 2010 Apr 23;5(4):e10294. doi: 10.1371/journal.pone.0010294 (PMC2859051; doi:10.1371/journal.pone.0010294)

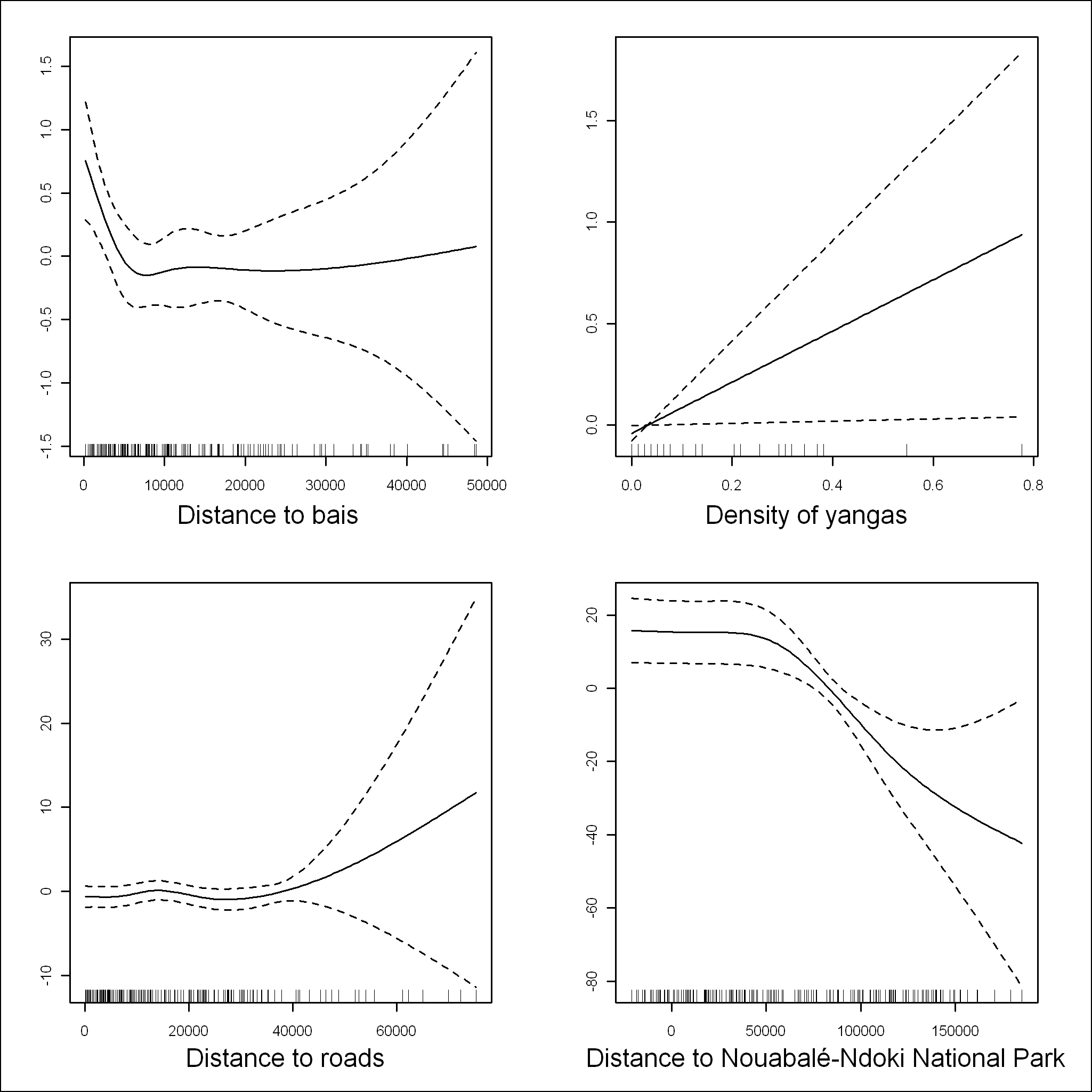

Supplement: Figure S1 — The composite model for elephant dung density. Estimated conditional dependence of elephant dung density on distance to bais, density of yangas, distance to roads and distance to the NNNP boundary. Estimates (solid lines) and confidence intervals (dashed lines), with a rug plot indicating the covariate values of observations (short vertical bars along each x-axis), are shown. Stratum and Y coordinate were also included as covariates. Note that y-axis scale is selected optimally for each covariate. (0.10 MB TIF) [file pone.0010294.s001.tif]

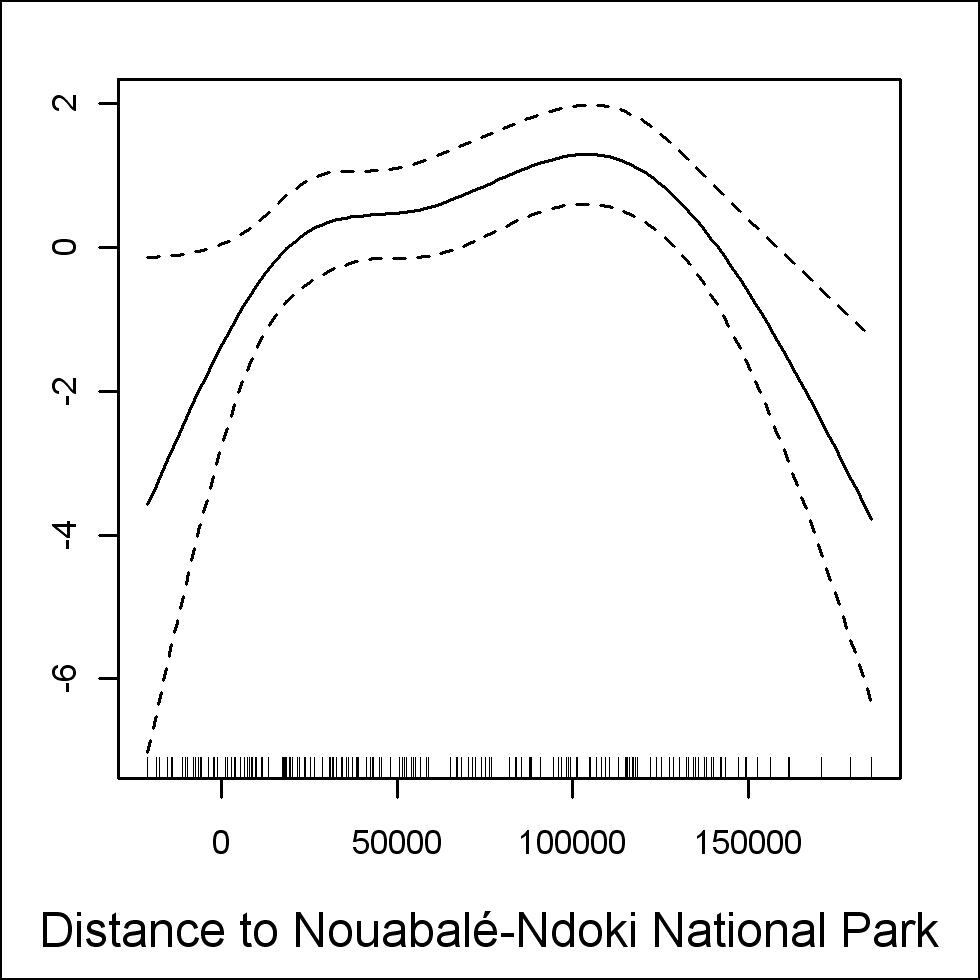

Supplement: Figure S2 — The composite model for gorilla nest density. Estimated conditional dependence of gorilla nest density on distance to NNNP boundary. Estimates (solid lines) and confidence intervals (dashed lines), with a rug plot indicating the covariate values of observations (short vertical bars along each x-axis), are shown. Stratum and X coordinate were also included as covariates. Note that all plots have the same y-axis scale. (0.03 MB TIF) [file pone.0010294.s002.tif]

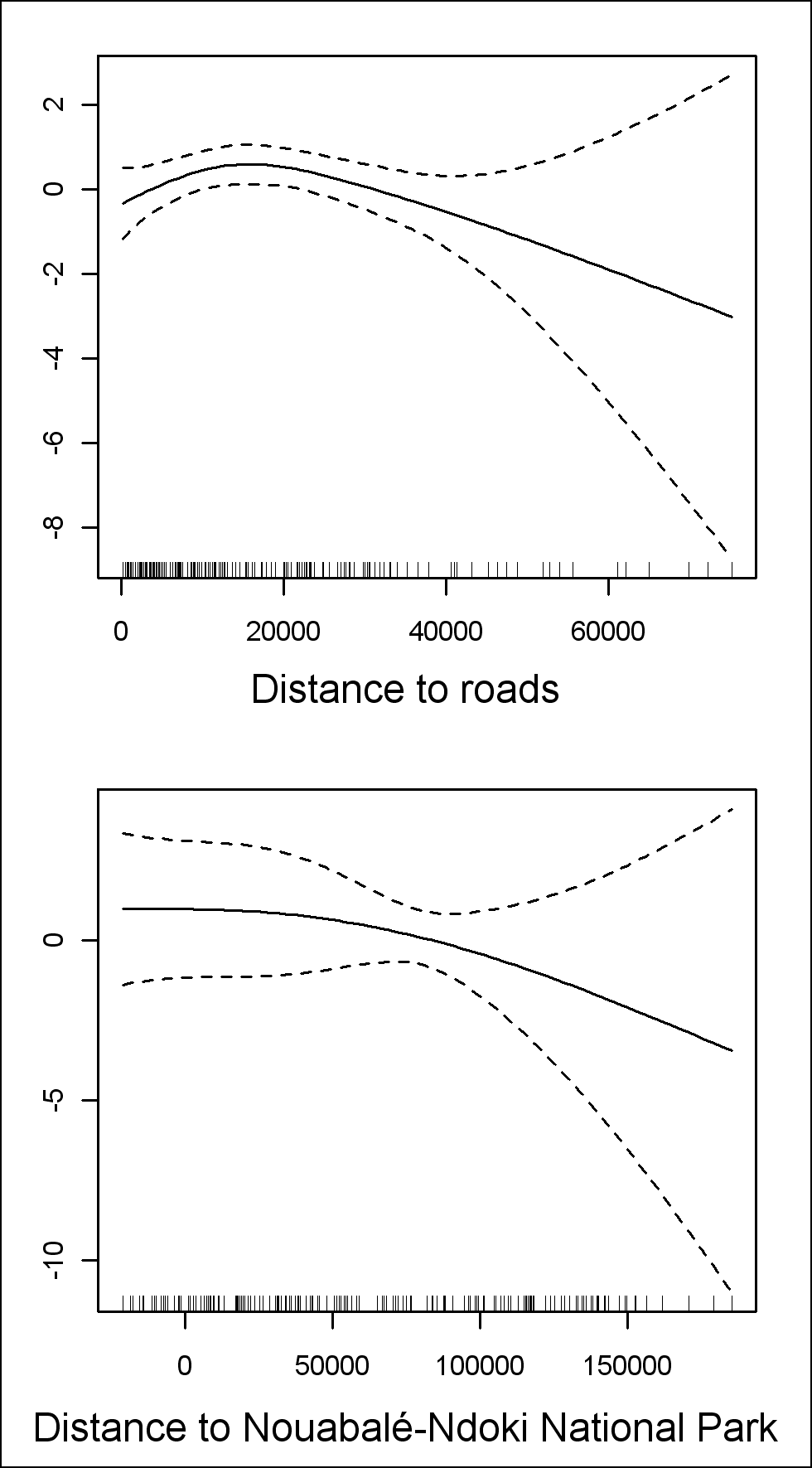

Supplement: Figure S3 — The composite model for chimpanzee nest density. Estimated conditional dependence of chimpanzee nest density on distance to roads and to the NNNP boundary. Estimates (solid lines) and confidence intervals (dashed lines), with a rug plot indicating the covariate values of observations (short vertical bars along each x-axis), are shown. Stratum and Y coordinate were also included as covariates. Note that all plots have the same y-axis scale. (0.05 MB TIF) [file pone.0010294.s003.tif]
